# A Hybrid Strategy Using Uterine Artery Embolization Followed by Hysteroscopic Morcellation for Vascular Retained Products of Conception After Spontaneous Miscarriage: Two Case Reports

**DOI:** 10.3390/jcm14196800

**Published:** 2025-09-26

**Authors:** Ryo Matsumoto, Kuniaki Ota, Takeshi Fukunaga, Kayo Tsuji, Yumiko Morimoto, Mika Sugihara, Yoshiaki Ota, Mitsuru Shiota, Koichiro Shimoya

**Affiliations:** 1 Department of Obstetrics and Gynecology, Kawasaki Medical School, Okayama 701-0192, Japank-tsuji@med.kawasaki-m.ac.jp (K.T.); y_morimoto@med.kawasaki-m.ac.jp (Y.M.); msugihara@med.kawasaki-m.ac.jp (M.S.); yoshimon@med.kawasaki-m.ac.jp (Y.O.); mshiota@med.kawasaki-m.ac.jp (M.S.); shimoya@med.kawasaki-m.ac.jp (K.S.); 2 Fukushima Medical Center for Children and Women, Fukushima Medical University, Fukushima 960-1295, Japan; 3 Department of Radiology, Kawasaki Medical School, Okayama 701-0192, Japan

**Keywords:** uterine artery embolization, hysteroscopic morcellation, retained products of conception, miscarriage, hysteroscopy

## Abstract

**Background/Objectives**: Retained products of conception (RPOC) are typically managed using dilation, curettage, or hysteroscopic resection. However, when the retained tissue is hypervascular, there is a significant risk of hemorrhage, particularly in cases of spontaneous miscarriage, in which vascular RPOC is rarely reported. Uterine artery embolization (UAE) is an established method for controlling acute bleeding. However, using mechanical hysteroscopic morcellation after UAE has not been fully explored. **Methods**: We report two cases of reproductive-aged women who developed vascular RPOC after spontaneous miscarriage, one following natural conception and the other following assisted reproduction. Both patients initially underwent expectant management but developed either acute or persistent vaginal bleeding. Imaging revealed hypervascular intrauterine lesions. UAE was performed using absorbable gelatin sponge particles targeting the ascending uterine artery branches. Following devascularization, hysteroscopic morcellation using the IBS or TruClear system was performed under direct visualization. **Results**: Intraoperatively, reddish vascular and whitish avascular degenerative tissues were noted. All retained tissues were completely resected with minimal bleeding. Both patients resumed menstruation shortly thereafter and expressed a desire for future pregnancy. **Conclusions**: This case series demonstrated the feasibility and effectiveness of a staged approach combining UAE and hysteroscopic morcellation for vascular RPOC management after spontaneous miscarriage. UAE improves surgical visibility and reduces bleeding risk, whereas mechanical morcellation ensures complete removal under direct vision with minimal trauma to the endometrium. This hybrid strategy may be a valuable fertility-preserving option, particularly in complex or hemodynamically unstable cases. Further prospective studies are needed to validate its safety, cost-effectiveness, and impact.

## 1. Introduction

The retention of placental or embryonic tissue within the uterine cavity, termed retained products of conception (RPOC), is a relatively common clinical entity observed following various pregnancy-related events, including vaginal delivery, cesarean section, spontaneous abortion, and elective termination [1,2]. The reported RPOC incidence is higher after medical abortions, with an incidence of 13% after vacuum aspiration and 29.4% after medical management [3]. Interestingly, RPOC incidence after spontaneous abortion is estimated to be very low (<1%); however, the reason is unknown [4]. The clinical presentation of RPOC varies, ranging from asymptomatic to life-threatening hemorrhage, particularly when the retained tissue is highly vascularized [5]. Management strategies for RPOC include expectant management, medical therapy, surgical evacuation (such as dilation, curettage, or hysteroscopic resection), and uterine artery embolization (UAE) [6,7]. Expectant management may be appropriate for stable patients with minimal symptoms and low vascularity, with spontaneous resolution reported in approximately 30% of cases [8]. If expectant management is ineffective, the first-line management typically involves uterotonics or surgical evacuation, such as dilation and curettage. However, when these procedures are difficult to perform in hemodynamically unstable patients with profound bleeding due to RPOC or when bleeding obscures the operative field, immediate intervention is often necessary to prevent hemodynamic instability [5,9]. UAE has emerged as a valuable fertility-preserving option for controlling acute hemorrhage in women with vascular RPOC, either as a definitive therapy or as a bridge to surgical intervention [9]. Notably, the combination of UAE followed by hysteroscopic resection is reportedly a safe and effective approach for the management of highly vascular RPOC, minimizing the risk of intraoperative hemorrhage and preserving reproductive potential [6,10].

Hysteroscopic removal allows for the selective treatment of RPOC under “visual control,” with minimal effects on the adjacent endometrium. This may lead to complete removal and reduced trauma, thereby decreasing the risk of inflammation, scarring, and intrauterine adhesions [10,11,12]. Accordingly, the number of reports on hysteroscopic resection for RPOC has been increasing [12,13,14,15,16]. More recently, hysteroscopic morcellation using a mechanical device has emerged as another option, offering rapid and precise tissue removal under direct vision [17,18]. However, a limitation of hysteroscopic approaches in general is that active bleeding within the uterine cavity can impair visibility. In such cases, preoperative UAE may improve visualization, thereby facilitating safer and more complete lesion removal. Despite these advantages, reports specifically describing the combined use of UAE and hysteroscopic morcellation remain limited [19], and no standardized protocol has yet been established for highly vascular RPOC.

Here, we report two cases of women who developed significant hemorrhage due to vascular RPOC following expectant management of a missed abortion. Both cases were successfully managed using a staged approach involving emergent UAE followed by hysteroscopic morcellation.

## 2. Materials and Methods

### 2.1. UAE Procedure

Pelvic angiography was initiated via the transfemoral approach under local anesthesia. A 4-French vascular sheath (Radifocus Introducer IIH; Terumo, Tokyo, Japan) was placed in the right common femoral artery, enabling subsequent catheterization and angiographic evaluation of the internal iliac arteries to delineate both uterine arteries and identify active bleeding sites. Bilateral uterine artery angiography was performed depending on vascular anatomy. To avoid injuring the vessel, a 2.0-F tip microcatheter (Carry Leon; UTM, Aichi, Japan) was used for selective cannulation of the uterine arteries. Embolization of the identified feeder vessels was then conducted using absorbable gelatin sponge particles (Serescue^®^; Astellas Pharma, Tokyo, Japan) at the level of the ascending branch of the uterine artery for fertility sparing. The completion of embolization was confirmed fluoroscopically by cessation of contrast flow and verified by repeat angiography of both uterine arteries.

### 2.2. Surgical Procedure

Hysteroscopic morcellation was performed by well-trained surgeons (IBS^®^ system, Intrauterine Bigatti Shaver, Karl Storz GmbH, & Co., Tuttlingen, Germany, or TruClear^TM^ 5C System, Medtronic, Minneapolis, MN, USA), which operated without electrical current, was employed; it utilized rotational cutting at 450 rpm in the IBS^®^ system or 800–1500 rpm in TruClear^TM^ 5C System with an aspiration flow of 500 mL/min under general anesthesia. The surgical procedure was performed as previously reported [20]. Briefly, cervical dilation was performed using a Hegar dilator up to a 6. Normal saline (NaCl 0.9%) was used as the distention medium. Tissue chips with blade number 6 (flute-beak shape) in the IBS^®^ system or a 3.1-mm blade (soft shaver mini) in TruClear^TM^ 5C System were used to remove RPOC at the same time as resection. After the IBS^®^ system or TruClear^TM^ 5C System was inserted into the uterine cavity and the pathological site was exposed and visualized, the rigid shaving device connected to the motor drive unit was introduced through the operative channel, and polyp resection was initiated. The resection began at the edge of the polyp and proceeded toward its base.

### 2.3. Case 1

**Figure 1:**
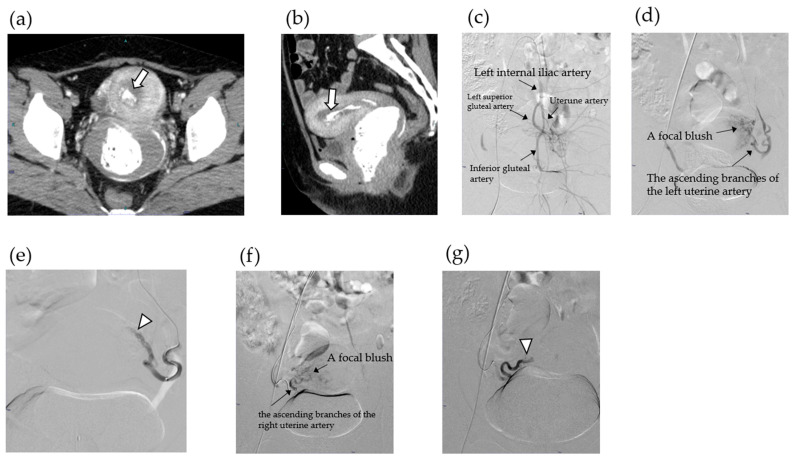
Arterial-phase contrast-enhanced computed tomography showing focal enhancement (arrow) within the endometrium in the axial (**a**) and sagittal (**b**) planes. Pelvic aortography showing the anatomy of the pelvic vessels (**c**) and the focal blush from the ascending branch of the left uterine artery (**d**). Post-embolization aortography showing contrast stasis without focal blush following complete embolization of the ascending branch of the left uterine artery using a gelatin sponge (arrowhead) (**e**). Pelvic aortography shows a focal blush from the ascending branch of the right uterine artery (**f**). Postembolization aortography showing contrast stasis without a focal blush following complete embolization of the ascending branch of the right uterine artery using a gelatin sponge (arrowhead) (**g**).

**Figure 2:**
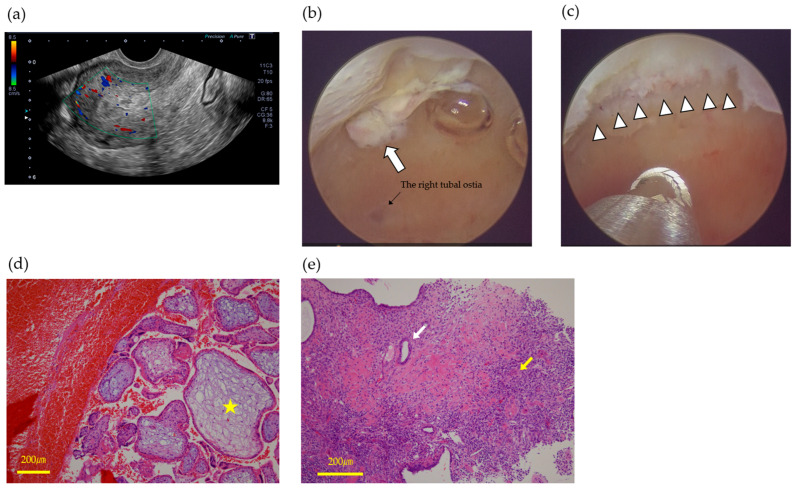
Transvaginal ultrasonography showing a hypovascular mass in the endometrium after uterine artery embolization@ (**a**). Hysteroscopic view of avascular and shirked retained products of conception (arrow) (**b**). By the methodology of IBS, the RPOC was completely removed without bleeding (arrowhead) (**c**). Chorionic villi with fibrotic/myxoid stroma, highlighted by yellow stars (**d**). Endometrial gland (white arrow) and surrounding stroma (yellow arrow), confirming the presence of endometrial components within the specimen (**e**).

A 36-year-old woman, gravida 5 para 2, with two spontaneous abortions (G5P2A2), conceived spontaneously and was diagnosed with missed abortion at 9 weeks of gestation during routine prenatal care. The patient opted for expectant management. Approximately 2 weeks later, she experienced a sudden onset of massive vaginal bleeding and was transported to the emergency department. On arrival, she was hypotensive (blood pressure 85/50 mmHg) and had tachycardia. Pelvic examination revealed a gestational sac partially protruding into the vagina, accompanied by approximately 900 g of blood clots. The patient’s hemoglobin level was 6.8 g/dL. Administration of uterotonics and hemostatic agents failed to achieve hemostasis, and intrauterine balloon tamponade was ineffective owing to ongoing active bleeding. Contrast-enhanced computed tomography (CT) demonstrated active arterial extravasation within the uterine cavity, consistent with highly vascular-RPOC (Figure 1a). Emergent bilateral UAE was performed using gelatin sponge particles to successfully control the hemorrhage (Figure 1b,c). The expelled tissue was pathologically confirmed as chorionic villi. The patient received blood transfusions and supportive care and was discharged in a stable condition 2 days later. Fourteen days post-UAE, transvaginal ultrasonography revealed a 16.9 × 11.8 mm intrauterine mass. Color Doppler imaging revealed no vascular flow. There was no evidence of uterine artery pseudoaneurysm (UAP) or arteriovenous malformation (AVM). The lesion was classified as type 0 RPOC according to the Gutenberg classification, which represents the evolution of Doppler-based characterization by incorporating both vascularity and echogenicity of ultrasound findings [21] (Figure 2a). Initial follow-up was conservative; however, 28 days after the UAE, the patient developed recurrent vaginal bleeding. Although her serum human chorionic gonadotropin (hCG) level was low (4.2 IU/mL), persistent bleeding necessitated further intervention. Hysteroscopic resection was performed using hysteroscopic morcellation on day 41 post-UAE. Intraoperatively, avascular, whitish, and degenerative tissues were observed and were completely resected (Figure 2b,c). Histopathology demonstrated chorionic villi (Figure 2d), accompanied by decidual and stromal tissue fragments (Figure 2e). The postoperative course was uneventful. The patient was discharged the following day, resumed regular menstruation, and expressed a desire for a future pregnancy.

### 2.4. Case 2

**Figure 3:**
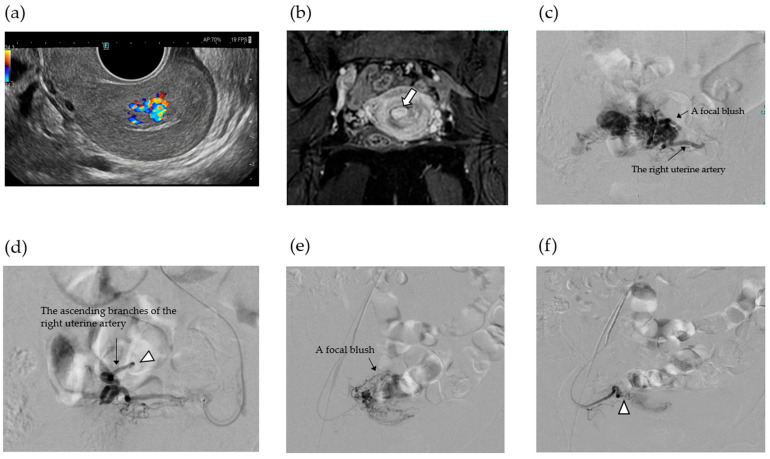
Transvaginal ultrasonography showing a hypervascular intrauterine mass (**a**). Sagittal contrast T2WI still demonstrated strongly contrasted retained products of conception (arrow) (**b**). Pelvic aortography showing a focal blush from the ascending branch of the left uterine artery (**c**). Post-embolization aortography showing contrast stasis without focal blush following complete embolization at the ascending branch of the left uterine artery using a gelatin sponge (arrowhead) (**d**). Pelvic aortography showing a focal blush from the ascending branch of the left uterine artery (**c**). Post-embolization aortography showing contrast stasis without focal blush following complete embolization at the ascending approximately 4 cm branch of the left uterine artery using a gelatin sponge (arrowhead) (**d**). Pelvic aortography shows a focal blush from the ascending branch of the right uterine artery (**e**). Postembolization aortography showing contrast stasis without a focal blush following complete embolization of the ascending branch of the right uterine artery using a gelatin sponge (arrowhead) (**f**).

**Figure 4:**
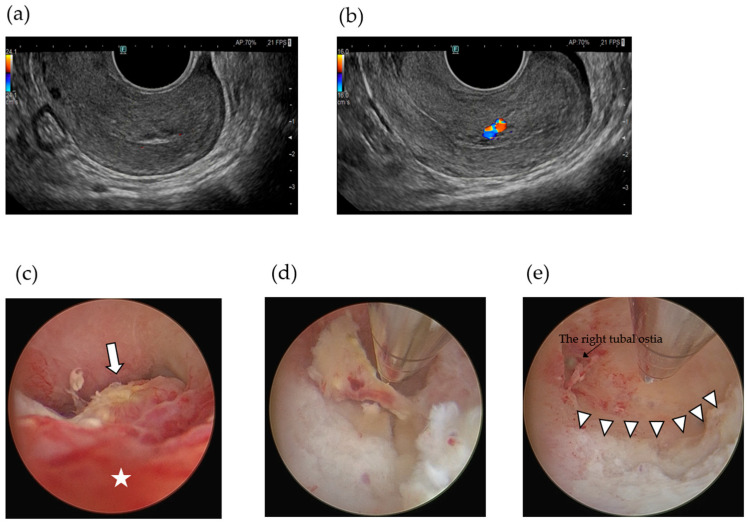
Transvaginal ultrasonography showing a hypovascular mass in the endometrium after uterine artery embolization (**a**). On day 17 after UAE, partial revascularization of the retained products of conception (RPOC) was observed on Doppler ultrasound (**b**). Hysteroscopic view showing two distinct areas of RPOC: a hyperemic, reddish lesion indicating revascularization (white star), and a whitish, shrunken area representing devascularized tissue (arrow) (**c**). Complete resection of the RPOC was achieved using a hysteroscopic morcellation system (triangles) (**d**,**e**).

**Figure 5:**
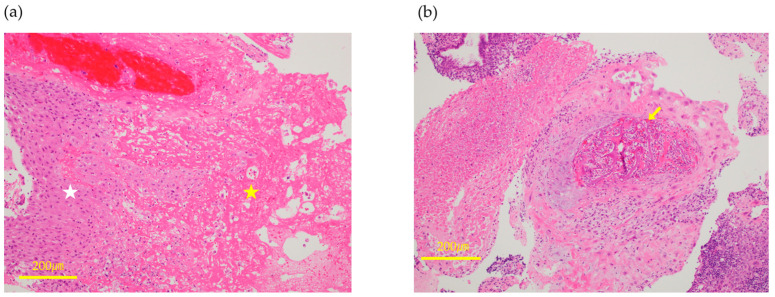
Histological section showing decidual tissue (white star) with fibrin deposition (yellow star), consistent with retained products of conception (**a**). Section of uterine artery specimen showing gelatin sponge embolic material (Serescue^®^) within the vascular lumen (yellow arrow), confirming successful embolization (**b**).

34-year-old woman, gravida 3 para 1, with two spontaneous abortions (G3P1A2) underwent frozen-thawed embryo transfer (FET) during a hormone replacement cycle; her serum hCG level was 594 mIU/mL 10 days after FET. Transvaginal ultrasonography revealed an intrauterine gestational sac (GS) at 5 + 3 weeks of gestation; however, she experienced spontaneous miscarriage at 6 + 2 weeks of gestation. One month after miscarriage, the patient began experiencing persistent menstrual-like genital bleeding; her serum hCG level remained elevated at 2780 mIU/mL, which represented a persistently high value without evidence of further decline, raising concern for trophoblastic persistence, and transvaginal ultrasound revealed a hypervascular intrauterine mass. The patient was referred to our institution for further evaluation and treatment. Transvaginal ultrasonography revealed a hypervascular intrauterine mass measuring 24 × 11 mm with some vascular flow extending into the myometrium (Figure 3a). Contrast-enhanced MRI confirmed that the lesion had invaginated the myometrium (Figure 3b). Based on these findings, a diagnosis of RPOC, Gutenberg type 3 with positive hypervascularity, was made [21]. Given the slow decline in the hCG levels and the need to exclude gestational trophoblastic disease, dilatation and evacuation (D&E) was considered. However, owing to the rich vascularity of the mass, we opted to perform UAE first, followed by hysteroscopic surgery as a staged procedure to embolize both uterine arteries (Figure 3c–f). On post-UAE day 1, her serum hCG level dropped to 577 mIU/mL, and Doppler ultrasound confirmed complete disappearance of the vascular flow within the mass measuring 15 × 7.0 mm (Figure 4a). The patient wished to become pregnant as soon as possible and requested urgent removal of the RPOC, with hysteroscopic morcellation scheduled for 18 days after UAE. On day 17 post-UAE, her serum hCG level declined to 31.5 mIU/mL, showing a steady decrease, which was attributed to the effect of UAE. Although partial revascularization of the RPOC was noted on preoperative Doppler ultrasound (Figure 4b), hysteroscopic morcellation was performed as scheduled on day 18. Intraoperatively, the retained tissue in the internal cervical os appeared reddish, suggesting residual vascularity, whereas the fundal lesion was whitish and avascular, consistent with ischemic changes (Figure 4c). Hysteroscopic morcellation was performed carefully (Figure 4d), and all retained tissue was completely removed (Figure 4e). The intraoperative bleeding was minimal. Histopathological examination revealed decidual tissue with fibrin deposition, but no definitive chorionic villi were identified (Figure 5a). These findings were consistent with RPOC, and there was no histological evidence suggestive of GTD. In addition, specimens obtained after uterine artery embolization demonstrated gelatin sponge embolic material (Serescue^®^; Astellas Pharma, Tokyo, Japan) within the vessel lumen, confirming the effectiveness of the embolization procedure (Figure 5b). The postoperative course was uneventful. The patient was discharged the following day, resumed regular menstruation, and expressed a desire for future pregnancy.

## 3. Discussion

RPOC is a common yet clinically heterogeneous condition, with presentations ranging from asymptomatic findings to severe, life-threatening hemorrhage, particularly in cases of hypervascular lesions. In both the cases presented in this report, massive or persistent uterine bleeding due to vascular RPOC was successfully managed using a staged approach combined with UAE, followed by hysteroscopic morcellation. This hybrid strategy was effective in achieving hemostasis while preserving fertility, demonstrating its utility in managing complex RPOC cases in which conventional curettage may pose significant risks.

Although dilation and curettage (D&C) has long been the standard surgical approach for RPOC, these risks, especially for women with future reproductive plans, are significantly greater in patients undergoing blind curettage [10,13,22,23]. In particular, in well-designed prospective study, D&C was associated with a high incidence of intrauterine adhesions [24]. Furthermore, Rein et al. [12] compared D&C with hysteroscopic resection and found that the incidence of postoperative intrauterine adhesions was 30.8% in the D&C group and 4.2% in the hysteroscopic resection group, with a significantly higher pregnancy rate in the hysteroscopic intervention group (*p* < 0.05) (68.8% vs. 59.9%). Previous systematic reviews have suggested that treatment of RPOC by curettage is associated with a higher incidence of intrauterine adhesions (IUAs) compared with hysteroscopic resection (29.6% vs. 12.8%) [10]. However, recent evidence from a multicenter randomized controlled trial reported a nonsignificant difference in IUAs between hysteroscopic morcellation (14.3%) and electric vacuum aspiration (20.6%), which does not fully align with the earlier systematic review [25]. Taken together, while hysteroscopic techniques—particularly morcellation—appear to reduce the risk of IUAs compared with blind curettage, further prospective trials are warranted to clarify the magnitude of this benefit. It is important to recognize that most individuals presenting with RPOC are of reproductive age and often have future fertility aspirations. Accordingly, management should aim not only for the thorough removal of the retained tissue but also for the preservation of endometrial and myometrial integrity to safeguard the reproductive potential [26]. Consequently, treatment for RPOC is shifting from blind D&C to visual hysteroscopic resection, and hysteroscopic morcellation may represent a promising approach that could evolve into a future standard of care.

Hysteroscopic techniques offer direct visualization, allowing precise and complete removal of the retained tissue with reduced endometrial trauma [27,28,29]. However, the use of electrosurgical energy during conventional resectoscopic procedures carries the inherent risk of thermal injury to adjacent tissues, including visceral structures [30]. Thermal spread may extend beyond the target lesion into the basal layer of the endometrium, potentially leading to healing through fibrosis rather than regeneration, thereby increasing the likelihood of intrauterine adhesion formation [31]. In contrast, mechanical hysteroscopic morcellation systems, such as the IBS^®^ device used in this study, eliminate the need for thermal energy, thereby avoiding the risk of heat-induced tissue damage. Furthermore, this technique employs a smaller diameter hysteroscope than traditional resectoscopes, requiring less cervical dilation and thereby reducing mechanical trauma [32]. Another advantage of hysteroscopic morcellation is its ability to maintain a consistently clear operative field through continuous tissue aspiration, thus minimizing the need for repeated scope insertion, shortening operative times, and reducing inadvertent endometrial injury [17,18,32]. On the other hand, conventional resectoscopic procedures are more commonly used for RPOC, whereas morcellation is less commonly described in RPOC management. In the current study, the patients expressed a desire to preserve their future fertility. Complete removal of the RPOC was successfully achieved using hysteroscopic morcellation, a fertility-sparing technique. Ongoing follow-up is warranted to assess long-term reproductive outcomes and potential perinatal complications.

One limitation of hysteroscopic surgery is its poor visibility in the presence of active intrauterine bleeding [33]. This is particularly problematic in vascular RPOC, in which profuse bleeding can obscure the operative field and increase the risk of incomplete resection. In this context, the UAE is a valuable adjunct. UAE enhances surgical visibility and reduces the risk of intraoperative hemorrhage by reducing vascular perfusion to the retained tissue. Additionally, the IBS has a good irrigation system; therefore, it is possible to maintain a clear view of the uterine cavity even with some bleeding. Therefore, combination therapy with UAE and IBS for RPOC with persistent bleeding may be the best treatment option for fertility preservation, as it improves the view of the uterine cavity. Kimura et al. reported that the technical and clinical success rates of UAE using a gelatin sponge for RPOC with hemorrhage were achieved without major complications in 93% and 100% of patients in the primary and second UAE [34]. Some studies have reported that, “if the size of the vascular RPOC is approximately 4 cm or less, conservative treatment with UAE alone may be possible [9,35]. However, Ko et al. concluded that prophylactic UAE before surgical interventions with a high risk of massive bleeding is safe and effective [36]. For cases of RPOC with marked vascularity, a combination approach including UAE to decrease blood flow, assessment with 3D color Doppler imaging, and subsequent hysteroscopic intervention may represent a feasible strategy [9]. In our present cases, the lesions measured 16.9 mm and 24 mm, respectively, and both responded well to the hybrid strategy. However, there is no consensus or evidence-based guideline regarding the optimal management of highly vascular RPOC, and prophylactic interventions should be considered with caution on a case-by-case basis. In our report, hysteroscopic morcellation was performed at 18 and 28 days after UAE. While the embolic effect may gradually diminish due to recanalization, significant reduction in vascularity often persists for several weeks, thereby creating a safe surgical window. In Case 1, decreased vascularity at day 28 enabled a safe and bloodless operative field. In Case 2, the patient strongly desired early removal to pursue pregnancy, and residual vascularity was still observed after UAE; thus, to prevent revascularization and further delay, surgery was scheduled at day 18. Both procedures were associated with minimal intraoperative bleeding, suggesting that UAE not only provides immediate hemostasis but also facilitates safe hysteroscopic intervention within a flexible timeframe. Accordingly, treatment should be individualized, balancing vascularity, timing, and patient preference. However, the optimal interval between UAE and hysteroscopic surgery remains undefined and warrants further study. Another advantage of this approach is its potential diagnostic yield for hysteroscopic morcellation. Currently available morcellators are equipped with an inbuilt suction apparatus that enables immediate removal of the resected tissue and facilitates the collection of all specimens for pathological examination [37]. In the present case, the resected tissue was avascular, fibrotic, and necrotic, consistent with nonviable RPOC. This contrasts with conventional curettage, in which tissue fragmentation and a lack of visual control may limit histopathological accuracy, particularly when differentiating RPOC from gestational trophoblastic disease.

To date, no report has described the combined use of UAE and hysteroscopic morcellation for the management of vascular RPOC. Our report provides a growing body of evidence supporting this strategy, along with a safe and effective alternative in carefully selected patients. However, this study had some limitations. First, UAE is not without risks, including potential ovarian dysfunction due to compromised collateral blood flow. In the present case, UAE targeting the ascending branches was performed using gelatin sponge particles to minimize the duration and extent of ischemia in the reproductive organs. Given the technical complexity of this procedure, the involvement of highly experienced interventional radiologists is essential to ensure its efficacy and safety. Second, the cost and availability of hysteroscopic morcellators may limit their widespread use, particularly in low-resource settings. However, we believe that there is a shorter learning curve and preprocedural setup compared to conventional hysteroscopic surgery, which might result in compensation for those costs due to the increasing number of cases in the future. Third, the long-term reproductive outcomes of this combined approach need to be elucidated and warrant further prospective studies.

## 4. Conclusions

In conclusion, staged use of UAE followed by hysteroscopic morcellation may represent a feasible and fertility-preserving option for managing vascular RPOC. This hybrid approach appears particularly advantageous in cases of massive or persistent bleeding, where conventional surgical evacuation may be limited by poor visibility or an increased risk of hemorrhage. By facilitating hemostasis and improving operative conditions, UAE enables precise and minimally invasive removal of residual tissue under direct visualization. Furthermore, mechanical morcellation minimizes endometrial trauma and preserves specimen integrity for accurate histopathological assessment. Notably, the present cases occurred after spontaneous miscarriage, a context in which vascular RPOC is rarely reported, underscoring the clinical relevance and novelty of this report. Although long-term reproductive outcomes remain to be fully elucidated, this strategy demonstrates promising potential, and further prospective studies are warranted to confirm its efficacy and establish standardized protocols.

## Data Availability

The data that support the findings of this study are available from the corresponding author, K.O., upon reasonable request.

## Abbreviations

The following abbreviations are used in this manuscript:

RPOC	retained products of conception
UAE	uterine artery embolization
